# Cost-Effectiveness of Pyrotinib Plus Capecitabine versus Lapatinib Plus Capecitabine for the Treatment of HER2-Positive Metastatic Breast Cancer in China: A Scenario Analysis of Health Insurance Coverage

**DOI:** 10.3390/curroncol29090476

**Published:** 2022-08-23

**Authors:** Yuwen Bao, Zhuolin Zhang, Xuan He, Lele Cai, Xiao Wang, Xin Li

**Affiliations:** 1 School of Health Policy and Management, Nanjing Medical University, Nanjing 211166, China; 2 School of Pharmacy, Nanjing Medical University, Nanjing 211166, China; 3 Center for Global Health, School of Public Health, Nanjing Medical University, Nanjing 211166, China

**Keywords:** cost-effectiveness, pyrotinib, lapatinib, partitioned survival model, metastatic breast cancer, health insurance coverage

## Abstract

Background: The overexpression of the human epidermal growth factor receptor-2 (HER2) gene is present in 20~25% of breast cancer (BC) patients, contributing to an inferior prognosis. Recent clinical trials showed that pyrotinib has promising antitumor activities and acceptable tolerability for those patients (ClinicalTrials.gov, NCT03080805 and NCT02422199). Therefore, this study aims to assess the cost-effectiveness of pyrotinib plus capecitabine versus lapatinib plus capecitabine for patients with HER2-positive metastatic BC after prior trastuzumab. Methods: A lifetime-partitioned survival model was established to evaluate health and economic outcomes with different treatment strategies. The primary outcome was the incremental cost-effectiveness ratio (ICER). Data were derived from the published literature, clinical trials, expert opinions, and other local charges. Sensitivity analyses were performed to assess the robustness of the findings. Scenario analyses were developed to make further evaluations. Results: The pyrotinib regimen had significant advantages over the lapatinib regimen after enrolling in the National Reimbursement Drug List (NRDL), with cost savings of USD 15,599.27 and a gain of 0.53 QALYs. Meanwhile, before enrolling in NRDL, the pyrotinib regimen afforded the same QALYs at a higher incremental cost of USD 45,400.64 versus the lapatinib regimen, producing an ICER of USD 85,944.79 per QALY. Scenario analyses yielded similar results. Sensitivity analyses suggested stability in the cost-effectiveness findings. Conclusions: Compared to lapatinib plus capecitabine, the pyrotinib plus capecitabine enrolled in NRDL is a cost-effective alternative second-line treatment for patients with HER2-positive metastatic BC in China.

## 1. Introduction

Breast cancer (BC) is one of the most common malignant tumors among women [1]. According to the global cancer burden data for 2020 released by the International Agency for Research on Cancer (IARC) of the World Health Organization (WHO), BC replaced lung cancer as the most often diagnosed cancer and ranked fifth as the leading cause of cancer mortality worldwide, with an estimated 2.3 million new cases (11.7%) and 685,000 deaths [2,3]. In China, the morbidity and mortality of female BC are rapidly increasing in recent years, accompanied by the trend that women are afflicted BC at a younger age [4]. Specifically, the number of incidence cases increased from 0.304 million in 2015 to 0.416 million in 2020, and the number of deaths increased by 47,174 over the five years, accounting for 17.1% of the global BC deaths [3,5]. The cancer burden has prominently grown over time in China, which can be attributed to diverse reasons, such as population aging, unhealthy lifestyles, and high body mass index [6,7,8].

Approximately 20~25% of the patients with BC demonstrate overexpression or amplification of human epidermal growth factor receptor 2 (HER2) [9,10], which is characterized by low chemosensitivity, strong invasion, high-risk recurrence, and poor prognosis [11,12,13]. This subtype leads to uncontrolled cancer cell regeneration. Despite the advent of HER2-targeted drugs, such as trastuzumab, pertuzumab, and trastuzumab emtansine (T-DM1), which have significantly improved health outcomes and the survival of patients [14,15], their resistance also inevitably develops [16]. In addition, some of the anti-HER2 agents are unavailable or unaffordable in some regions of the world [13]. Thus, improving anti-HER2 strategies for patients who are intolerant or experience failed standard first-line therapies is of importance.

Lapatinib is an orally reversible small molecule, belonging to the tyrosine kinase inhibitor (TKI) family of the target domain of epidermal growth factor receptor (EGFR) and HER2 [17]. It has been listed as a treatment for HER2-positive BC in China for several years. An economic evaluation based on NeoALTTO and BCIRG 006 trials considered that combining lapatinib with paclitaxel, cyclophosphamide, and trastuzumab (TCH) would be cost-effective as an adjuvant regimen versus TCH [18]. Conversely, another pharmacoeconomic study reported that the addition of lapatinib to capecitabine had no noticeable benefits compared to capecitabine monotherapy, which displayed an ICER that exceeded willingness-to-pay threshold limits [19].

To incorporate valuable new drugs into the Basic Medical Insurance Drug List and to improve the accessibility of drugs in a timely manner, the Chinese government adjusted Medicare Drug Lists through price negotiations with the drug manufacturer. After the national medical insurance price negotiation in 2018, 97 innovative drugs were included in the Type B category of the National Drugs Catalogue of Basic Medical Insurance, Industrial Injury Insurance and Reproductive Insurance (2019 edition), which included pyrotinib, with drug prices cut by an average of 60.7% [20]. Pyrotinib is a second-generation irreversible pan-HER tyrosine kinase inhibitor that potently targets EGFR, HER2, and HER4 [21,22]. It is also an innovative drug developed independently by an indigenous pharmaceutical company (Jiangsu Hengrui Medicine Co., Ltd., Lianyungang, China) in China for the treatment of HER2-positive metastatic BC [12]. Recent clinical trials and real-world studies demonstrated that pyrotinib has a profound effect on clinical efficacy and safety for patients who had previously received trastuzumab [23]. For example, in a phase Ⅰ study, pyrotinib achieved an overall response rate of 83.3%, with a median progression-free survival (PFS) of 8.85 months in trastuzumab-naïve patients [24]. Another double-blind, randomized phase Ⅲ study (PHENIX) indicated that pyrotinib combined with capecitabine significantly prolonged median PFS compared with capecitabine monotherapy. The results showed that the median PFS was 11.1 months versus 4.1 months in pyrotinib versus placebo groups, respectively [25]. Two open-label, multicenter, randomized controlled trialsin phases Ⅱ and Ⅲ were based on the Chinese population. Both of them discovered that pyrotinib plus capecitabine has improvied efficacies than lapatinib plus capecitabine, with longer PFS (18.1 months vs. 7.0 months in phase Ⅱ trial and 12.5 months vs. 6.8 months in phase Ⅲ trial, respectively) and higher overall response rates (78.5% vs. 57.1% in phase Ⅱ trial and 67% vs. 52% in phase Ⅲ trial, respectively) after prior trastuzumab [26,27], thereby mutually confirming findings. Simultaneously, the results of the real-world study were consistent with clinical trials, proving that pyrotinib is beneficial for HER2-positive metastatic BC patients by maintaining an excellent clinical benefit rate of 87.5% [28]. The most frequent grade 3 or worse adverse events (AEs) reported in the above were diarrhea and hand-foot syndrome [26], which were generally well tolerated and easily manageable.

Both pyrotinib and lapatinib belong to tyrosine kinase inhibitors, which are similar in structure and target sites. They are standard treatment drugs recommended by the Breast Cancer Guidelines of Chinese Society of Clinical Oncology (CSCO) [29,30], and they are also suitable for second-line treatments of advanced HER2-positive BC after tolerance of trastuzumab. Background characteristics were comparable between the two drugs. In China, recommended optional drugs for the second-line treatment of HER2-positive metastatic BC were scarce. Trastuzumab emtansine (T-DM1), another guideline recommended drug, is an antibody-drug conjugate. Its mechanism of action is different from that of pyrotinib and lapatinib. At the same time, there have been international pharmacoeconomic studies on T-DM1 [31]; thus, in our study, it is excluded.

Currently, there were several clinical trials on the efficacy and safety of pyrotinib and lapatinib, and it has been proven that pyrotinib has superior clinical efficacy than lapatinib. However, the research on pharmacoeconomics between two drugs is lacking and few pharmacoeconomic studies have been conducted on anti-HER2 second-line agents globally. In addition, the impact of the national medical insurance price negotiation on the economics of certain innovative anti-cancer medicine in China is unknown. Therefore, we hypothesized that the national drug price negotiation reduced the price of pyrotinib, thus increasing the cost-effectiveness of the treatment containing this innovative anti-cancer medicine without modifying its clinical efficacy. The aims of our study were as follows: (1) to estimate the cost-effectiveness of pyrotinib plus capecitabine versus lapatinib plus capecitabine in patients with previously treated HER2-positive metastatic BC from the perspective of the Chinese healthcare system; and (2) to validate the above-mentioned hypothesis that the national medical insurance price negotiation could pose positive impact on the economics of pyrotinib.

## 2. Materials and Methods

### 2.1. Patients and Treatment

Our research was based on the outcomes of published clinical trials, which were registered as ClinicalTrials.gov, NCT03080805 and NCT02422199 [26,27]. The demographic characteristics of the two trials were similar. In general, eligible patients were 18 to 70 years of age, had pathologically confirmed HER2-positive relapsed or metastatic BC, and had previously been treated with trastuzumab. They all received up to two prior lines of chemotherapy and they were 50-year-old on average.

The treatment strategy consisted of two different regimens: (1) pyrotinib plus capecitabine, including whether pyrotinib was enrolled in the National Reimbursement Drug List (NRDL) [32] or not, which is referred as the pyrotinib-A regimen and pyrotinib-B regimen for distinguishment purposes. More concretely, the former represented that pyrotinib was not included in NRDL, while the latter was covered by NRDL, and the only difference between the two regimens was the price of pyrotinib. (2) The second regimen comprises lapatinib plus capecitabine. Both treatment regimens received oral capecitabine 1000 mg/m^2^ twice daily on days 1 to 14 of each cycle combined with pyrotinib at a dose of 400 mg orally daily or lapatinib at a dose of 1250 mg orally daily. The mean weight and body surface area used to calculate dosing were reflective of Chinese patients. Patients continued with their respective regimens until progression. Because the clinical data were obtained from the published report of clinical trials, ethical approval was not required for the study on participants in accordance with the local legislation and institutional requirements.

### 2.2. Model Structure

**Figure 1:**
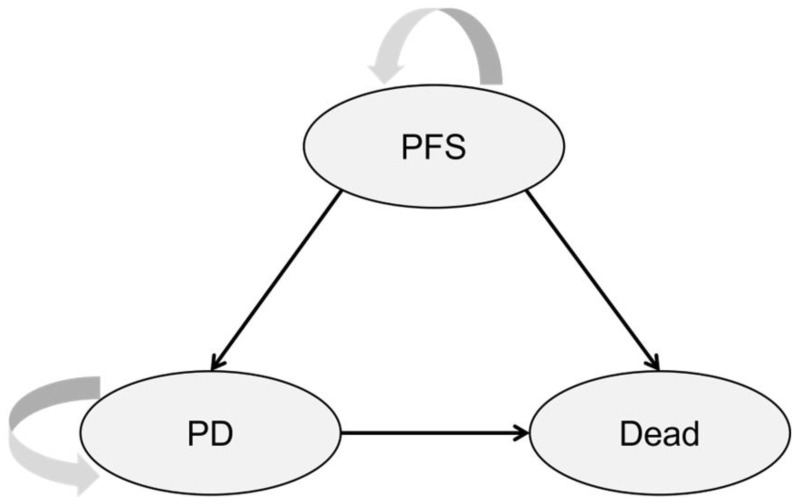
Partitioned survival model for HER2-positive metastatic breast cancer patients. Abbreviations: PFS, progression-free survival; PD, progressive disease; HER2, human epidermal growth factor receptor 2.

**Figure 2:**
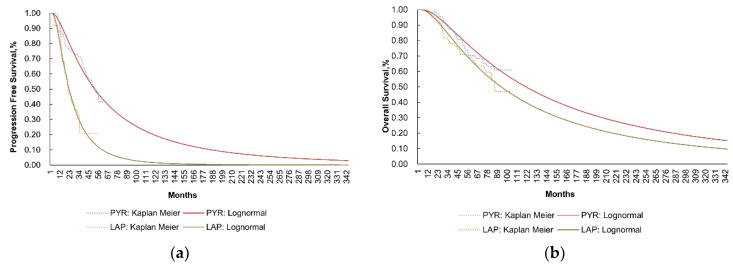
Kaplan–Meier Curve and Lognormal-estimated progression-free survival (**a**) and overall survival (**b**) with pyrotinib plus capecitabine and lapatinib plus capecitabine. Abbreviations: PYR, pyrotinib; LAP, lapatinib.

We constructed a partitioned survival model (PSM) in Microsoft Excel 2016 (Microsoft, Redmond, WA, USA) to analyze the cost-effectiveness of the pyrotinib plus capecitabine regimen compared to the lapatinib plus capecitabine regimen for patients with HER2-positive metastatic BC after prior trastuzumab. This economic model is executed from the perspective of the Chinese healthcare system. It comprises three mutually exclusive health states, which are presented in Figure 1: progression-free survival (PFS) state, progressive disease (PD) state, and death as the terminal state. PFS was the baseline at which all patients entered the model. From this state, patients could remain stable or experience progression and enter PD or develop to the death stage. From the PD state, patients continued to progress or die. The death stage was an absorption state, including various disease-related or unrelated demise. Survival curves for PFS and OS were employed in the model to directly reckon state occupancy over time without the need for transition probabilities between health states. The area under the PFS curve figured the proportion of patients who were alive with no disease progression and the area under the OS curve figured the proportion of patients who were alive. The departure between OS and PFS provided the number of patients who were alive with progressed disease (Figure 2). The OS data of the PHOEBE clinical trial were immature, and due to the characteristics of the population in the NCT02422199 trial, the data had high similarities with those in PHOEBE; the PFS and OS curves in the NCT02422199 trial were adopted. Half-cycle corrections were applied to rectify both costs and QALYs.

The length of each model cycle was set at 3 weeks (21 days), and the time horizon was set to be a lifetime in this model, referring to the previous study [26,27]. The primary outcome was presented as the incremental cost-effectiveness ratio (ICER), which was defined as the cost of gaining one QALY. Based on the guidelines for the Evaluations of Pharmacoeconomic in China [33], a 1 to 3 times account for the per capita gross domestic product (GDP) in 2021 was taken as the willingness-to-pay (WTP) threshold, which ranged from 12,552.90 to 376,58.70 USD/QALY [34]. The monetary values in Chinese Yuan Renminbi (RMB) were converted into United States dollars (USD) based on the average exchange rate in 2021 (1 RMB = 0.15502 USD). We then converted the annual discount rate of 5% into the discount rate of each cycle for application.

### 2.3. Cost Data

Considering the perspective of the Chinese healthcare system, only direct medical costs were adopted in this model, incorporating the costs of drugs, AE treatments, subsequent treatment, and regular laboratory examination. All costs were reported at the 2021 price levels in USD and adjusted on the medical care consumer price index (CPI).

The drug treatment costs were mainly incurred by pyrotinib, lapatinib, and capecitabine. The usage and dosage of three drugs were consistent with the instructions. According to the unit drug price obtained from the hospital drug database and their usage and dosage, the costs of drug treatment were calculated. The AE treatment costs, which not only contained agents but also included hospitalization expenses, were calculated through expert consultation of eight BC clinicians from different hospitals. Subsequent treatment costs were calculated according to the follow-up treatment program and its proportion of the PHOEBE clinical trial, combined with clinical expert opinions and the average bid-winning prices of required therapeutic drugs in various provinces of China in 2021. Furthermore, laboratory examination costs, including blood routine tests, urine routine tests, liver, kidney function tests, and so on, were calculated based on the actual investigation cost of various inspection items in hospitals. Patients were monitored once a cycle until death. Drug doses were calculated for a standard female body surface area of 1.62 m^2^ with a relative body weight of 59 kg based on the report of Nutrition and Chronic Disease Status of Chinese Residents (2020) [35]. An overview of the cost data is shown in Table 1.

### 2.4. Adverse Events

The analysis included grade 3 or worse AEs. According to the published literature, the most common grade 3 to 4 AEs of patients with HER2-positive metastatic BC were diarrhea and hand-foot syndrome, which mainly occurred during the first and second cycles of treatment [26]. Therefore, this study assumed that AEs only occur in the first two cycles, and the treatment costs of AEs would not be included later. The probability of patients experiencing Grade ≥ 3 AEs was derived from the findings of a phase Ⅲ (PHOEBE) clinical trial (Table A1).

### 2.5. Utility Values

Economic outcomes were expressed by QALYs combined with health-state utility values with the time spent in each health state. Major health state utility scores were derived from relevant published forms of literature based on the Chinese population, using the scale of EQ-5D [36]. The utility values estimated for the PFS and PD states were 0.740 and 0.468, respectively, and they were the same between pyrotinib and lapatinib groups. Disutility values linked with AEs, mainly containing diarrhea and hand-foot syndrome, were also stemmed from the literature and corrected by the proportion in clinical data [26,37]. The utilities and related data were summarized in Table 1.

### 2.6. Sensitivity Analysis

One-way sensitivity and probabilistic sensitivity analyses were performed to explore the uncertainty of the model. In one-way sensitivity analyses, only one variable would be altered at a time. The maximum and minimum prices of capecitabine were taken from the highest or lowest centralized purchase price in 2021 (Table A2 and Table A3). The maximum and minimum value of costs of subsequence treatments were obtained through clinical consultation. Due to the lack of a 95% confidence interval value, the variable of other costs fluctuated around 20% of the base value, while the range of utility scores fluctuated by 10% based on the baseline. According to the guidelines, the maximum and minimum annual discount rates were recommended to be set at 8% and 0%, respectively [33], so that the upper and lower limit values of discount rates for each cycle in this analysis were separately calculated to be 0.44% and 0%, respectively. The influence of variables on ICERs was illustrated in the tornado graph, presented in descending order. In probabilistic sensitivity analysis, costs were placed as the gamma distribution, and the utility value varied with the beta distribution, adopting 1000 Monte Carlo simulations to evaluate the impact of model variables by extracting the values of the corresponding distribution. The outcomes of probabilistic sensitivity analysis were exhibited using a scatter plot of 1000 iterations and the cost-effectiveness acceptable curve (CEAC).

## 3. Results

### 3.1. Base Case Analysis

Over a lifetime, the results of the base case analysis of two treatment regimens are expressed by total costs, total QALYs, and ICERs. Taking the lapatinib regimen as a reference, the ICERs were calculated by dividing the incremental costs by the incremental effects. The calculation formula is shown in Equation (1).

(1) $$\mathrm{ICER}=\frac{\Delta {\mathrm{Cos}t}_{\mathrm{pyrotinib}}-\Delta {\mathrm{Cos}t}_{\mathrm{lapatinib}}}{\Delta {\mathrm{QALY}}_{\mathrm{pyrotinib}}-\Delta {\mathrm{QALY}}_{\mathrm{lapatinib}}}$$

The pyrotinib-B regimen, which meant being enrolled in NRDL, conferred a cost saving of USD 15,599.27 and an additional gain of 0.53 QALYs. Lower costs and higher QALYs were more cost-effective than the lapatinib regimen, which indicated that the pyrotinib-B regimen not only reduced the heavy disease burden but also improved the quality of life for cancer patients. On the other hand, before being enrolled in NRDL, the pyrotinib-A regimen also provided an additional 0.53 QALYs but with a higher incremental cost of USD 45,400.64 compared with the lapatinib treatment, leading to an ICER of USD 85,944.79 per QALY. It was far beyond the WTP threshold of three-times GDP per QALY in China (37658.7USD/QALY). Pyrotinib-B plus capecitabine was considered to be superior to lapatinib plus capecitabine; simultaneously, the pyrotinib-A regimen was not a cost-effective strategy. More details are shown in Table 2.

### 3.2. Sensitivity Analysis

#### 3.2.1. One-Way Sensitivity Analysis

**Figure 3:**
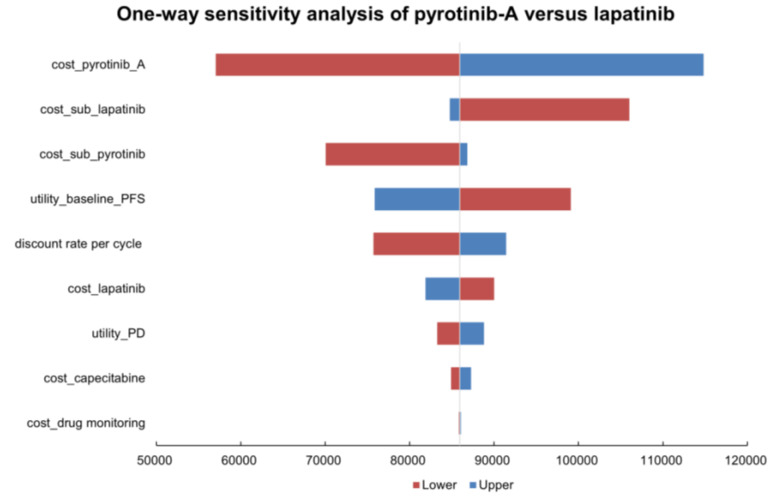
Tornado diagram for one-way sensitivity analysis of pyrotinib-A plus capecitabine versus lapatinib plus capecitabine. Abbreviations: pyrotinib-A, pyrotinib before being enrolled in National Reimbursement Drug List; PFS, progression-free survival; PD, progressive disease; sub, subsequent.

**Figure 4:**
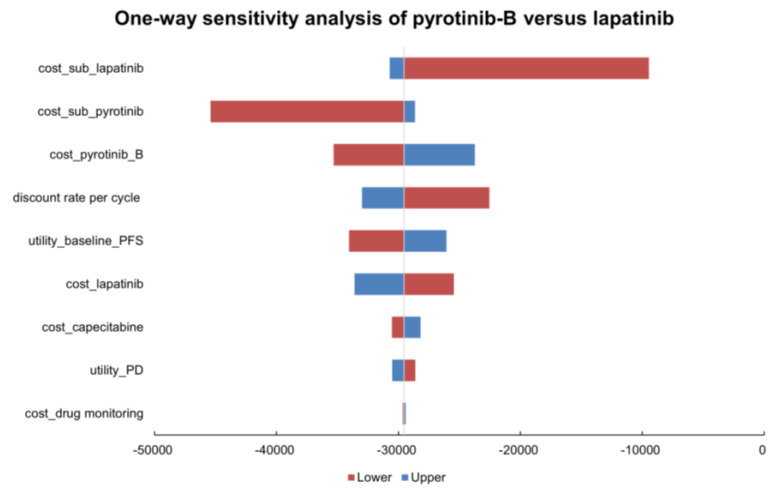
Tornado diagram for one-way sensitivity analysis of pyrotinib-B plus capecitabine versus lapatinib plus capecitabine. Abbreviations: pyrotinib-B, pyrotinib after being enrolled in National Reimbursement Drug List; PFS, progression-free survival; PD, progressive disease; sub, subsequent.

To determine variables that have greater impacts on findings, one-way sensitivity analyses were executed. As shown in Figure 3, the results of the one-way sensitivity analysis between pyrotinib-A and lapatinib regimens were exhibited by tornado diagrams, revealing the costs of pyrotinib-A and the costs of subsequent treatments, and the utility of PFS affected the model most. Figure 4, the one-way sensitivity analysis between pyrotinib-B and lapatinib regimens manifested that the results were sensitive to the costs of subsequent treatments, pyrotinib-B, and discount rate per cycle. Overall, subsequent treatment costs and pyrotinib costs play important roles in the robustness of the model.

#### 3.2.2. Probability Sensitivity Analysis

**Figure 5:**
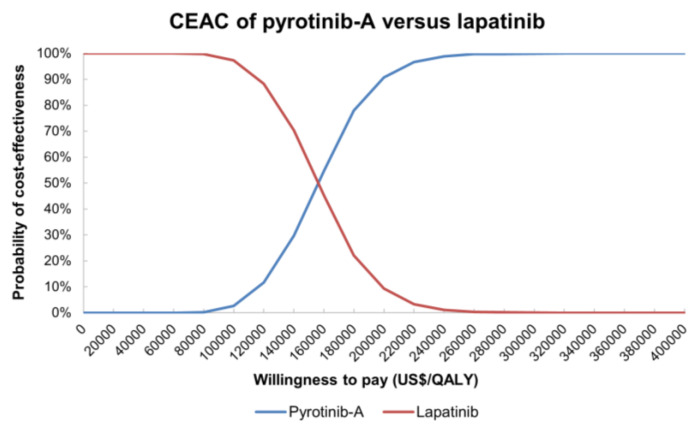
Cost-effectiveness acceptability curve between pyrotinib-A plus capecitabine and lapatinib plus capecitabine. Notes: Blue line indicates pyrotinib-A regimen, and red line indicates lapatinib regimen. Abbreviations: CEAC, cost-effectiveness acceptability curve; pyrotinib-A, pyrotinib before being enrolled in National Reimbursement Drug List; QALY, quality-adjusted life-year.

**Figure 6:**
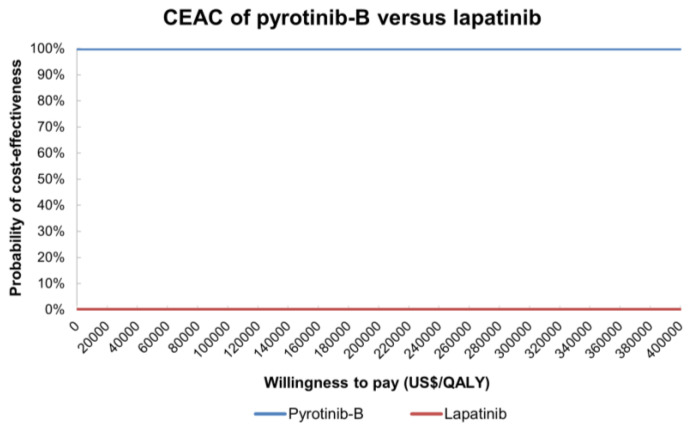
Cost-effectiveness acceptability curve between pyrotinib-B plus capecitabine and lapatinib plus capecitabine. Notes: Blue line indicates pyrotinib-B regimen, and red line indicates lapatinib regimen. Abbreviations: CEAC, cost-effectiveness acceptability curve; pyrotinib-B, pyrotinib after being enrolled in National Reimbursement Drug List; QALY, quality-adjusted life-year.

**Figure A1:**
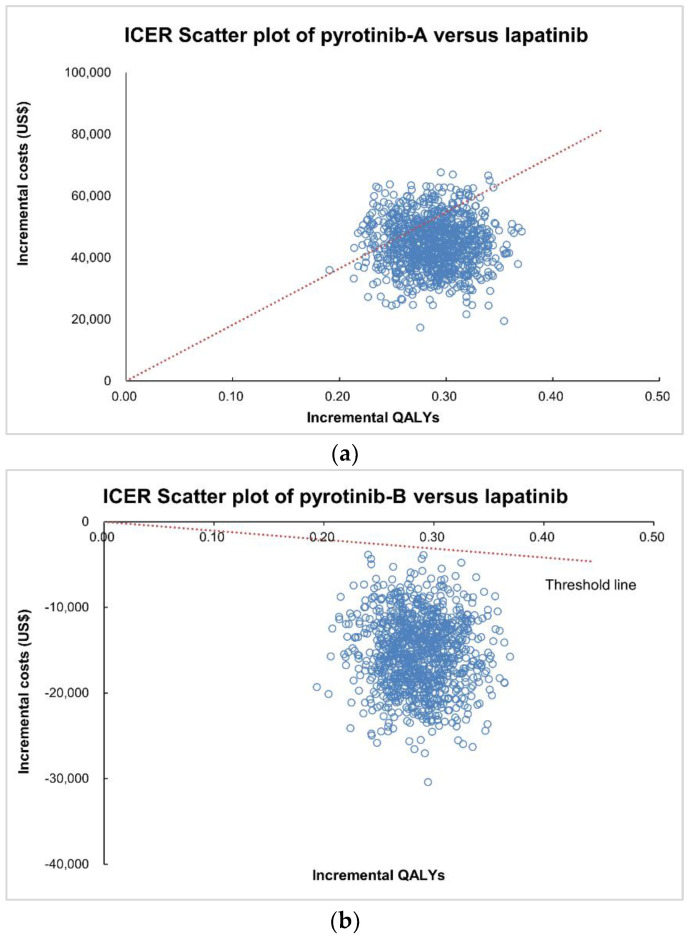
ICER Scatter plot. (**a**) ICER Scatter plot of pyrotinib-A plus capecitabine versus lapatinib plus capecitabine; (**b**) ICER Scatter plot of pyrotinib-B plus capecitabine versus lapatinib plus capecitabine. Abbreviations: ICER, Incremental cost-effectiveness ratio; pyrotinib-A, pyrotinib before being enrolled in the National Reimbursement Drug List; pyrotinib-B, pyrotinib after being enrolled in the National Reimbursement Drug List.

The incremental cost-effectiveness scatters plot (Figure A1) and the cost-effectiveness acceptability curve (CEAC) of the target population were obtained through a Monte Carlo simulation of 1000 times. The CEAC on pyrotinib-A regimen versus lapatinib regimen indicated that at a WTP threshold of no more than USD 157,000 per QALY, the probabilities of the pyrotinib-A regimen as a cost-effectiveness option were 0% (Figure 5). Separately, at a threshold of USD 160,000 per QALY and USD 170,000 per QALY, 79.7% and 99.4% of simulations generated a result in which the pyrotinib-A regimen was more cost-effective than the lapatinib regimen. CEAE associated with the result of probabilistic sensitivity analyses on pyrotinib-B and lapatinib (Figure 6) demonstrated that the pyrotinib-B regimen was always keeping advantages over the lapatinib regimen.

### 3.3. Scenario Analysis

#### 3.3.1. Scenario A: Time Horizon

The setting of 5-year and 10-year time horizons yielded similar results to the base case analysis. Specifically, the average survival time of BC patients was 27 years in China, and the 5-year and 10-year time horizons approximated one-fifth and one-third of the lifetime range. Both 5-year or 10-year were shorter observation times than a lifetime; however, the results still indicated that the pyrotinib-B regimen dominated the lapatinib regimen, generating fewer costs as well as higher efficiency.

#### 3.3.2. Scenario B: Price Adjustment

In the setting where pyrotinib was not enrolled in NRDL, our findings revealed that the price of pyrotinib-A dropping approximately 40% could improve the cost-effectiveness of the second-line treatment of pyrotinib plus capecitabine versus lapatinib plus capecitabine under the condition of constant QALYs, gaining an ICER of USD 28159.31 per QALY. When the price of pyrotinib-A diminished by 60%, the pyrotinib-A regimen would be economically attractive and its ICER was below one-fold the per capita GDP, displaying the preferred strategy. In the setting where pyrotinib was enrolled in NRDL, the lapatinib regimen would not be cost-effective even if lapatinib had 100% price reduction according to the results of the comparison between pyrotinib-B and lapatinib regimens. The results of the scenario analysis were condensed in Table 3.

## 4. Discussion

Metastatic BC remains a devastating disease with a poor prognosis and limited treatment options. Pyrotinib, a Chinese innovative anti-cancer drug approved in China in 2018 [38], has profound significance for patients with HER2-positive metastatic BC who are resistant to trastuzumab. Based on pivotal clinical trials, this study conducted the cost-effectiveness analysis of pyrotinib plus capecitabine versus lapatinib plus capecitabine. It demonstrated that the pyrotinib regimen had prominently advantages over the lapatinib regimen after enrolling in NRDL from the perspective of the Chinese healthcare system. On the other hand, before the inclusion of pyrotinib in NRDL, ICER was far more than three-times the GDP per capita. In other words, adding pyrotinib to capecitabine as a combination therapy for the second-line treatment of HER2-positive metastatic BC was less likely to be cost-effective at the generally acceptable WTP threshold in 2021. The probability sensitivity analysis showed the robustness of our model and findings. It was only when the threshold of WTP was set at USD 157,000 per QALY did the pyrotinib strategy before enrolling in NRDL could have a 57% probability of being economically attractive versus the lapatinib strategy. This threshold exceeded the willingness to pay. Therefore, the price of the lapatinib regimen could not be considered cost-effective enough. The scenario analysis associated with the expense of pyrotinib revealed that pyrotinib plus capecitabine compared with the lapatinib strategy was the preferred regimen when the drug costs declined by 40%. The scenario analysis related to the 5-year and 10-year time horizons produced similar outcomes, with pyrotinib-B yielding fewer costs and higher QALYs. Obviously, health insurance coverage is a support to reduce the burden of patients. Moreover, in the long term, it is conducive to promote patients in returning to societal life.

There are two randomized, controlled second-line clinical trials for trastuzumab-refractory patients based on pyrotinib versus lapatinib in the Chinese population [26,27], which are similar in demographic characteristics, establishing the clinical evidence of the efficacy and safety of pyrotinib. These trials demonstrated that pyrotinib has superior clinical effects. More concretely, compared with lapatinib, the incidence of AEs of pyrotinib decreased, showing improved safety. In addition, it can prolong PFS and improve the quality of life of patients to some extent. Patients suffering from metastatic BC would inevitably delay work due to treatment, resulting in a loss in productivity. At the same time, psychological changes brought by the disease are often exported with negative emotions, leading to a reduction in work efficiency and the deterioration of interpersonal relationships. From the societal perspective, a longer PFS means that patients can better maintain their working ability and output labor values more effectively, thus improving all social benefits. It will be a virtuous circle.

Despite the fact that clinical trials of second-line BC treatments are not rare, seldom consistent standards and treatment options of second-line anti-HER2-positive BC drugs are recommended by clinical experts globally. Thus, it is not difficult to find that second-line therapeutic drugs exhibit a lot of development space in the treatment of HER2-positive BC. Trastuzumab has been proven to be a specific drug for HER2-positive BC in first-line treatments [38,39]; simultaneously, it was easy to inevitably cause resistance and cardiotoxicity [40,41]. The addition of pertuzumab could alleviate the tolerance of trastuzumab. Unfortunately, despite the clinical efficacy reported in several studies, relevant pharmacoeconomic analyses have failed to confirm the addition of pertuzumab to trastuzumab and docetaxel (THP) as first-line treatments for patients with HER2-positive metastatic BC are cost-effective compared with standard therapy options (trastuzumab and docetaxel, TH) [10,42]. In an incurable setting, an improved PFS means more time spent accruing costs for expensive therapies. It explained that even after a 100% price reduction at the conventional threshold, the pertuzumab regimen was still unlikely to provide reasonable economic value and likely to produce an additional healthcare burden [20,43]. It further confirmed the necessity of investigating second-line therapeutic drugs.

Several previous studies have shown notable clinical efficacy for second-line continuing treatments with trastuzumab-containing (T-DM1) therapies after disease progression. However, the results varied widely according to treatment usage as well as the national reimbursement policy. For instance, T-DM1 had benefits in Canada compared with trastuzumab monotherapy depending on the results of economic evaluation [44]. On the contrary, a cost-effectiveness analysis by Zhang et al. indicated that T-DM1 was not a cost-effective option as a second-line therapy in China and ought to conduct appropriate price reduction [31].

Accordingly, this comparative study of the second-line drugs pyrotinib and lapatinib is of innovative implication and practical value. Pharmacoeconomic effects of lapatinib have been explored abroad, suggesting that the treatment with lapatinib plus capecitabine could be considered a cost-effective alternative to the continuation of trastuzumab in patients who have progressed on trastuzumab in the metastatic setting [45]. Nonetheless, the economic evaluation was not the latest achievement and cannot represent the current situation.

Up until now, no pharmacoeconomic evaluation studies on pyrotinib have been developed worldwide. Our research was groundbreaking and it has implications for understanding the pharmacoeconomic value of pyrotinib, which were consistent with our research hypothesis. Considering the significant changes in pyrotinib’s price before and after price negotiations, the distinct advantages of its inclusion in NRDL for patients was further confirmed. It also provided evidence and guidance for the national negotiation of drug price renewal in reverse, and it supports medical insurance decisions in China. Specifically, in some other developed countries or areas such as the United States and the European Union, pyrotinib has not yet been approved for marketing. To some extent, this study furnished a theoretical basis and reference for accelerating domestic drugs to be marketed in other countries.

While this study provides relevant insights into the economy and efficacy of pyrotinib in the Chinese population, some limitations deserve to be noted. Firstly, since the phase 3 (PHOEBE) clinical trial was attached with an immature OS curve, the establishment of the partitioned survival model was referred to in two similar clinical trials. PFS and OS data were obtained from the phase 2 trial (NCT02422199) and the other data such as demography and AEs were derived from the PHOEBE trial. Although having high similarities in disease types, population groups, and screening, both of them still inescapably excited some bias that affected outcomes. Secondly, the disutility value of AEs was uncertain. In this study, the disutility of AEs of HER2-positive metastatic BC was analyzed by calibrating the disutility value in a previous UK study according to the proportion of AEs in clinical trials. The baseline utility value was based on the EQ-5D scale for metastatic BC in China. It is difficult to obtain accurate utility scores for the Chinese population with HER2-positive metastatic BC. Finally, although pyrotinib is one of the recommended therapeutic drugs for advanced BC patients in China, it has not been widely used all over the world. The analysis of this study was only based on the Chinese population, lacking evidence from other populations; thus, there was no comparison between BC patients from different countries. More research needs to be carried out to obtain detailed data.

## 5. Conclusions

We performed a PSM-based economic evaluation to access the cost-effectiveness of adding pyrotinib to capecitabine as a combination therapy for patients with HER2-positive metastatic BC after prior trastuzumab. Compared with lapatinib plus capecitabine, the pyrotinib regimen after being enrolled in NRDL is undoubtedly a cost-effective alternative second-line treatment for targeting the population in China. Moreover, our findings showed that pyrotinib before enrolling in NRDL was not an economically attractive option. Due to the lack of clinical trials in other groups, the economic benefits of the pyrotinib regimen on populations in other countries need to be further verified. Furthermore, decision makers ought to take into account the maintenance of economic advantages of the pyrotinib strategy.

## Figures and Tables

**Table 1:** Summary of base-case values in PSM.

Variables	Base-Case Values	Upper Limit	Lower Limit	Standard Error	Distribution	Reference
Costs						
Pyrotinib-A	2895.00	3474.00	2316.00	272.94	Gamma	hospital
Pyrotinib-B	580.93	697.12	464.74	54.77	Gamma	hospital
Lapatinib	1085.03	1302.04	868.03	102.30	Gamma	hospital
Capecitabine	62.32	105.48	29.65	17.93	Gamma	hospital
Subsequent_PYR	1070.50	1087.61	767.24	84.96	Gamma	expert opinions, [26]
Subsequent_LAP	1506.61	1524.87	1194.82	87.45	Gamma	expert opinions, [26]
AEs_PYR	62.99	75.58	50.39	5.94	Gamma	expert opinions, [26]
AEs_LAP	30.4	36.48	24.32	2.87	Gamma	expert opinions, [26]
AEs_Hospitalization	123.83	148.59	99.06	11.67	Gamma	hospital, [26]
Drug monitoring	33.56	40.27	26.84	3.16	Gamma	hospital, [26]
Health-state utility values						
PFS	0.740	0.814	0.666	0.0349	Beta	[36]
PD	0.468	0.515	0.421	0.0221	Beta	[36,37]
AE disutility values						
Diarrhea (PYR + LAP)	0.051	0.056	0.045	0.0024	Beta	[26,37]
Hand-foot (PYR + LAP)	0.027	0.029	0.024	0.0012	Beta	[26,37]
Discount rate, %						
Discount rate	5.00%	8.00%	0.00%	0.0191	Normal	[33]

Notes: Costs are represented in USD. The data listed in the table are all values in one cycle. Dosage: pyrotinib 400 mg once daily; lapatinib 1250 mg once daily; capecitabine 1000 mg/m^2^, day 1–14/per cycle, twice daily. Female body surface area: 1.62 m^2^/59 kg. Abbreviations: PSM, Partitioned Survival Model; Pyrotinib-A, pyrotinib before being enrolled in National Reimbursement Drug List; pyrotinib-B, pyrotinib after being enrolled in National Reimbursement Drug List; PYR, pyrotinib; LAP, lapatinib; AEs, adverse events.

**Table 2:** Summary of total and incremental costs and QALYs of PYR-A + CAP vs. LAP + CAP, PYR-B + CAP vs. LAP + CAP, among HER2-positive metastatic BC patients.

Table	Costs (USD)	QALYs	Incremental Costs	Incremental QALYs	ICER (US$/QALY)
PYR-A + CAP ^1^	109726.16	1.86	45400.64	0.53	85944.79
PYR-B + CAP ^2^	48726.25	1.86	−15599.27	0.53	−29529.90
LAP + CAP	64325.52	1.33			

Notes: ^1^ PYR-A + CAP versus LAP+CAP; ^2^ PYR-B + CAP versus LAP + CAP. Half-cycle correction was used for the results. Abbreviations: PYR-A + CAP, pyrotinib-A plus capecitabine, means pyrotinib before being enrolled in National Reimbursement Drug List; PYR-B + CAP, pyrotinib-B plus capecitabine, means pyrotinib after being enrolled in National Reimbursement Drug List; LAP, lapatinib; QALY, quality-adjusted life-year; ICER, incremental cost-effectiveness ratio.

**Table 3:** Scenario analysis results in price reductions relative to pyrotinib and lapatinib.

Price Adjustment	ICERs (PYR-A vs. LAP)	ICERs (PYR-B vs. LAP)
10%	71,498.44	−27,497.92
20%	57,052.07	−25,465.97
30%	42,605.69	−23,434.02
40%	28,159.31	−21,402.07
50%	13,712.94	−19,370.12
60%	Dominant	−17,338.17
70%	Dominant	−15,306.22
80%	Dominant	−13,274.27
90%	Dominant	−11,242.32

Abbreviations: PYR-A, pyrotinib before being enrolled in National Reimbursement Drug List; PYR-B, pyrotinib after being enrolled in National Reimbursement Drug List; LAP, lapatinib; ICERs, incremental cost-effectiveness ratios.

## Data Availability

The detailed data are contained within the article: Appendix A and Appendix B. Further inquiries can be directed to the corresponding author.

## Appendix A

**Table A1:** Grade 3 or worse adverse events and its proportion between pyrotinib plus capecitabine and lapatinib plus capecitabine of patients with HER2-positive metastatic breast cancer.

AEs, (Grade ≥ 3)	Proportion of PYR + CAP, %	Proportion of LAP + CAP, %	Reference
Diarrhea	30.6	8.3	[26]
Hand-foot syndrome	16.4	15.2	[26]
Vomiting	6.0	2.3	[26]
White blood cell count	7.4	0.8	[26]
Neutrophil count decreased	7.5	3.8	[26]

Notes: The proportion of grade 3 or worse AEs exceeding 10% was taken into account in this study. Abbreviations: AEs, adverse events; PYR, pyrotinib; LAP, lapatinib; CAP, capecitabine.

**Table A2:** The maximum and minimum input values of one-way sensitivity analysis between pyrotinib-A plus capecitabine and lapatinib plus capecitabine.

Variables (Range)	Maximum ICER	Minimum ICER
cost_drug monitoring (26.84 to 40.27)	86,066.0394	85,808.4278
cost_capecitabine (29.65 to 105.48)	87,282.3157	84,918.7577
utility_PD (0.421 to 0.515)	88,811.8946	83,258.0974
cost_lapatinib (868.03 to 1302.04)	81,873.6848	90,000.7823
discount rate per cycle (0.0044 to 0)	91,416.5327	75,715.0284
utility_baseline_PFS (0.666 to 0.814)	75,870.0543	99,106.3971
cost_sub_pyrotinib (767.24 to 1087.61)	86,833.6547	70,047.7306
cost_sub_lapatinib (1194.82 to 1524.87)	84,761.6212	106,008.2993
cost_pyrotinib_A (2316.00 to 3474.00)	114,827.4397	57,047.0274

Abbreviations: ICER, Incremental cost-effectiveness ratio; PFS, progression-free survival; PD, progressive disease; sub, subsequent; pyrotinib-A, pyrotinib before being enrolled in the National Reimbursement Drug List.

**Table A3:** The maximum and minimum input values of one-way sensitivity analysis between pyrotinib-B plus capecitabine and lapatinib plus capecitabine.

Variables (Range)	Maximum ICER	Minimum ICER
cost_drug monitoring (26.84 to 40.27)	−29,398.4940	−29,656.1056
utility_PD (0.421 to 0.515)	−30,515.0088	−28,606.7715
cost_capecitabine (29.65 to 105.48)	−28,182.2177	−30,545.7757
cost_lapatinib (868.03 to 1302.04)	−33,590.8486	−25,463.7511
utility_baseline_PFS (0.666 to 0.814)	−26,068.3030	−34,052.1120
discount rate per cycle (0.0044 to 0)	−32,983.6944	−22,545.7296
cost_pyrotinib_B (464.74 to 697.12)	−23,730.0004	−35,324.5993
cost_sub_pyrotinib (767.24 to 1087.61)	−28,630.8787	−45,416.8028
cost_sub_lapatinib (1194.82 to 1524.87)	−30,702.9120	−9456.2341

Abbreviations: ICER, Incremental cost-effectiveness ratio; PFS, progression-free survival; PD, progressive disease; sub, subsequent; pyrotinib-B, pyrotinib after being enrolled in the National Reimbursement Drug List.
